# Development of rearing methodology for the invasive Spotted Lanternfly, *Lycorma delicatula* (Hemiptera: Fulgoridae)

**DOI:** 10.3389/finsc.2022.1025193

**Published:** 2022-09-21

**Authors:** Laura J. Nixon, Sharon Jones, Andrew C. Dechaine, Dalton Ludwick, Mauri Hickin, Liam Sullivan, Johanna E. Elsensohn, Juli Gould, Melody Keena, Thomas Kuhar, Douglas G. Pfeiffer, Tracy C. Leskey

**Affiliations:** ^1^ United States Department of Agriculture - Agricultural Research Service (USDA - ARS), Appalachian Fruit Research Station, Kearneysville, WV, United States; ^2^ Department of Entomology, Virginia Tech, Blacksburg, VA, United States; ^3^ Department of Entomology, Texas A&M AgriLife Research & Extension Center, Corpus Christi, TX, United States; ^4^ Forest Pest Methods Laboratory, USDA APHIS PPQ S&T, Buzzards Bay, MA, United States; ^5^ Graduate Interdisciplinary Program in Entomology and Insect Science, The University of Arizona, Tucson, AZ, United States; ^6^ Northern Research Station, USDA Forest Service, Hamden, CT, United States

**Keywords:** Lycorma delicatula, colony, Ailanthus altissima, rearing, phloem-feeding

## Abstract

*Lycorma delicatula*, White (Hemiptera: Fulgoridae), spotted lanternfly, is a univoltine, phloem-feeding, polyphagous and invasive insect in the USA. Although a primary host for this species is *Ailanthus altissima*, tree of heaven, *L. delicatula* also feeds on a wide range of hosts important to the USA including cultivated grapevines. Due to the need for classical or augmentative biological control programs to reduce impacts of *L. delicatula* across invaded areas, we developed a laboratory-based rearing protocol for this invasive species. Here, we evaluated the use of *A. altissima* apical meristems, epicormic shoots, and fresh foliage cut from *A. altissima* as a food source for rearing newly hatched *L. delicatula*. On these sources of plant material <20% of *L. delicatula* developed into adults and no oviposition occurred. However, when young, potted *A. altissima* trees were used as a food source, >50% of *L. delicatula* nymphs developed to the adult stage under natural daylengths and temperatures ranging from 20–25°C. The addition of wild grapevine, *Vitis riparia*, did not increase survivorship or reduce development time. To elicit mating and oviposition, adults were provided with *A. altissima* logs as an oviposition substrate and maintained under shortened daylengths and reduced nighttime temperatures (12L:12D and 24°C:13°C). This resulted in 2.12 egg masses deposited per female, which was 4× more than when adults were maintained in standard rearing conditions (16L:8D and 25°C). Based on these experiments, we present a protocol for reliably rearing *L. delicatula* under laboratory and/or greenhouse conditions.

## Introduction

*Lycorma delicatula* White (Hemiptera: Fulgoridae), spotted lanternfly, is an invasive planthopper first detected in the USA in Berks County, PA in 2014 (1, 2). *Lycorma delicatula* has continued to spread and establish populations across Eastern states (3). *Lycorma delicatula* is univoltine with four nymphal instars; first instar nymphs emerge from overwintered egg masses in the spring. Nymphs develop throughout the late spring and summer and begin to emerge as adults in July (4, 5). Adult populations feed heavily in the late summer and reproduce throughout fall, generally dying off during hard frosts (4, 5).

*Lycorma delicatula* is a polyphagous phloem feeder with over 100 host plants reported globally (6). Tree of heaven, *Ailanthus altissima* (Mill.) Swingle (Sapindales: Simaroubaceae), is often referred to as the primary or preferred host of *L. delicatula*, although it is not obligatory for completion of their development (7–9). Damage caused by *L. delicatula* phloem feeding has been reported on grapevine and peach trees in its invaded range in South Korea (4). Commercial vineyards in PA, USA have suffered losses despite rigorous insecticide regimes for this pest (4, 10). Recent studies on *L. delicatula* feeding effects on young peach trees, point to an increase in frost damage susceptibility after *L. delicatula* infestation (11). *Lycorma delicatula* also causes nuisance problems and indirect plant damage as they produce large amounts of honeydew as they feed, which coats vegetation, enabling growth of black sooty mold (4).

Eradication efforts have focused on *A. altissima* management and removal (12), with longer term solutions targeting biological control agents (13–16). Indeed, an egg parasitoid *Dryinus sinicus* Olmi (Hymenoptera: Dryinidae), and a nymphal parasitoid *Anastatus orientalis* Yang & Choi (Hymenoptera: Eupelmidae), both from *L. delicatula*’s native range, are being evaluated for suitability within the USA as part of a classical biological control program. Rearing parasitoids, however, requires a continuous supply of the appropriate lifestage of the target host.

As *L. delicatula* is univoltine, establishing and maintaining a productive colony can be challenging. Information on such variables as diapause during the egg stage and nutritional needs for L. delicatula is still emerging (7–9, 17). Here, we evaluated several sources of *A. altissima* plant material and abiotic conditions promoting *L. delicatula* development and survivorship and different substrates for the promotion of mating and oviposition under laboratory conditions to generate standardized methods for maintaining a colony of *L. delicatula*. Our goal was to develop rearing methodology that is feasible and flexible for a range of research and biological control programs.

## Materials and methods

### Field collection of *Lycorma delicatula*

*Lycorma delicatula* egg masses for rearing studies conducted in quarantine facilities in Fort Detrick, MD and Blacksburg, VA were collected from host trees in a quarantine zone in Winchester, VA (within a 1-mile radius of 39°12’40.5”N 78°09’18.3”W). Sections of tree bark or branches harboring egg masses detached from trees were carefully handled and sized to fit into sealed Ziploc bags which were subsequently placed in sealed coolers and transported to quarantine greenhouses in accordance with APHIS permits P526P-18-03369 and P526P-18-02138, respectively. Additionally, nymphs and adult *L. delicatula* were collected from *A. altissima* in Winchester, VA, placed in mesh cages which were sealed in coolers, and transported to Fort Detrick, MD, where a quarantine greenhouse was used for additional rearing-related studies in accordance with APHIS permit P526P-18-03369. Insects for used for rearing studies from 2016-2018 at the Forest Pest Methods Laboratory, were collected from infested sites in Berks County, PA. Egg masses and the bark containing the mass were carefully chipped from host trees, placed in plastic boxes with mesh for ventilation. Boxes were double contained in a sealed 50 gallon barrel or sealed cooler, for transport to Buzzards Bay, MA. The egg masses were taken into quarantine, separated, dried in a laminar flow hood and stored in an environmental chamber (5̊ 0:0 (L:D) 65% RH) until they were removed and used for rearing studies. All collections were in accordance with APHIS permits P526P-17-04376.

### *Lycorma delicatula* development and survivorship on *Ailanthus altissima* diet preparations

**Figure 1:**
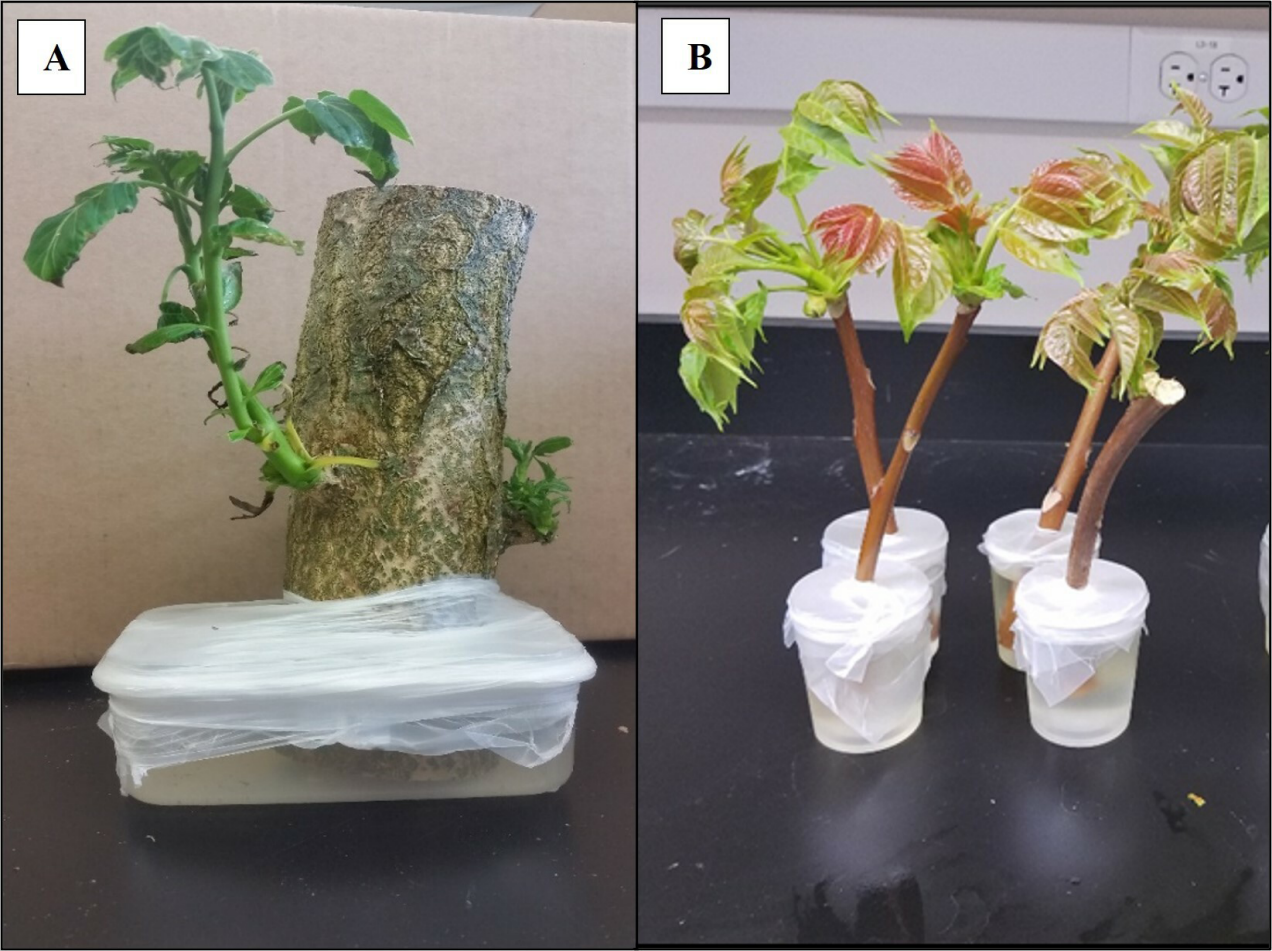
**(A)** Bolt of A altissima producing epicormic shoots in a container of fertilizer. **(B)** Apical meristem bolts of A altissima with foliage in a container of fertilizer. Photo credit: Mauri Hickin.

Three *A. altissima* plant diet materials were evaluated: 1) epicormic shoots generated on bolts of *A. altissima >*5* cm* diam and placed in water and Maxi-Gro (General Hydroponics, Santa Rosa, CA) until shoots emerged ~ 4 weeks later; 2) apical meristems generated on bolts <5 cm diam and placed in water and Maxi-Gro to promote shoot and foliage propagation ~4 weeks later and 3) freshly cut branches from *A. altissima* in the field that included full leaves and woody stems (<5 cm diam) placed in water and Maxi-Gro. For rearing trials, plant material was cut to 50 cm in length, immediately placed in a container of water and Maxi-Gro and sealed in place using Parafilm (Bemis Company Inc., Neenah, WI) to prevent insects drowning in the water source (see **Figures 1A, B**). Two containers of each type of plant material (epicormic shoots, apical meristems or freshly cut branches) were placed in separate cages (W32.5 × D32.5 × H77.0 cm, 680 µm aperture mesh, BugDorm-4S3074 Insect Rearing Cage, MegaView Science Co., Taiwan) and newly hatched (<72 h) *L. delicatula* nymphs were introduced. Survivorship and development were recorded every 2-3 d until adulthood, and plant material was changed as needed, generally once per week. All trials were conducted in quarantine facilities at USDA-APHIS, Buzzards Bay, MA (2016-2018), USDA-ARS, Fort Detrick, MD (2019), and Virginia Tech., Blacksburg, VA (2019). Across all locations five cages of containing epicormic shoots, seven cages containing apical meristems, and ten cages containing freshly cut branches were evaluated; all cages had starting numbers of 20 – 50 nymphs per cage.

Additionally, a preliminary trial evaluating the possibility of rearing *L. delicatula* nymphs on young, potted *A. altissima* was also conducted at Fort Detrick, MD in 2019. Newly hatched nymphs were placed in cages containing *A. altissima* (<1 year old, ~30 cm tall) trees. While nymphal starting numbers, survivorship, and developmental stage were not recorded during this pre-trial, the number of adults produced was documented.

### *Lycorma delicatula* development and survivorship on potted *A. altissima* and *V. riparia* plant diets

In 2020, a study using potted plants based on 2019 preliminary trial was conducted. *Ailanthus altissima* trees were grown from seeds extracted from samaras collected in the field in October 2019. Samaras were stratified in a refrigerator at 5 – 7°C for 60 – 90 d, after which the wings were removed from seeds, and seeds then allowed to soak in water at room temperature (~22°C) for 18 h. Seeds were then planted into flat trays containing 5 cm deep potting mix (Pro-mix Premier BK25 Mycorrhizae, Premier Horticulture Inc., Quakertown, PA), and placed in a growth chamber (25°C, 16:8) to germinate. After 4 weeks, seedlings were transplanted into 6.5 L pots and moved to a greenhouse for maintenance with optimal conditions being 22 - 25°C with 16:8 L:D. Additionally, native grapevine, *Vitis riparia* Michx. (Vitales: Vitaceae), was purchased (Cold Stream Farm, Free Soil, MI) and planted into 0.6 L pot with potting soil. *V. riparia* vines were maintained at a length <50 cm under greenhouse conditions described above until use in rearing studies.

**Figure 2:**
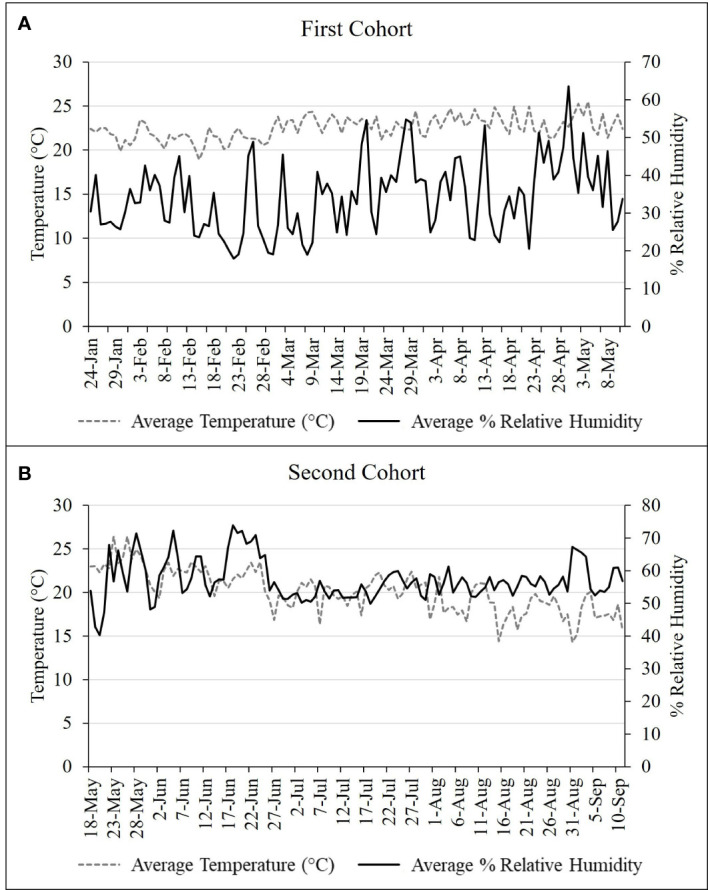
Daily temperature and humidity readings for **(A)** first and **(B)** second nymphal cohort study conducted in 2020 from January – May and May –September, respectively.

Two cohorts of nymphs were used. The first cohort included nymphs that emerged from eggs collected in Northampton and Lehigh counties, PA in October 2019, held at a constant 15°C until hatch in January 2020. The second cohort was comprised of eggs collected in Winchester, VA in February 2020, held at a constant 10°C for 2 months, and then at 25°C for 2-3 additional weeks until hatch in May 2020. For the first cohort, 43 1^st^ instar nymphs and for the second cohort, 27 mixed 1^st^ and 2^nd^ instar nymphs were introduced per cage. Cages (W32.5 × D32.5 × H77.0 cm, 680 µm aperture mesh, BugDorm) contained either a single *A. altissima* in a 6.5 L pot or an *A. altissima* in a 6.5 L pot and a *V. riparia* plant in a 0.6 L pot. Tracking development of the first and second cohorts began 24 January and 18 May 2020, respectively. All trials were conducted in a quarantine greenhouse at Fort Detrick, Frederick, MD (see **Figures 2A, B** for environmental conditions) under natural daylengths supplemented with strip lighting (T-5 High-Output Fixture - 54 W 2-Lamp, FarmTek, Dyersville, IA) set to 16L:8D. For each cohort, four cages containing *A. altissima* alone and four cages containing *A. altissima* plus *V. riparia* were assessed for survivorship and development of *L. delicatula* every 3 – 4 d. Plants were changed as needed, approximately every three weeks during 1^st^ – 3^rd^ nymphal instars and every two weeks thereafter. Once 4^th^ instars molted to adults, they were transferred to corresponding cages held in an environmental chamber to monitor mating and oviposition.

Data from the first and second cohorts were analyzed separately, and all statistical analyses were performed using JMP software v.16.0 (SAS Institute, Cary, NC, USA). For each cohort, the effect of diet on the amount of time spent in each lifestage and total development period for *L. delicatula* were analyzed using an independent two-sample t-test.

### Conditions Necessary to Elicit Oviposition

**Figure 3:**
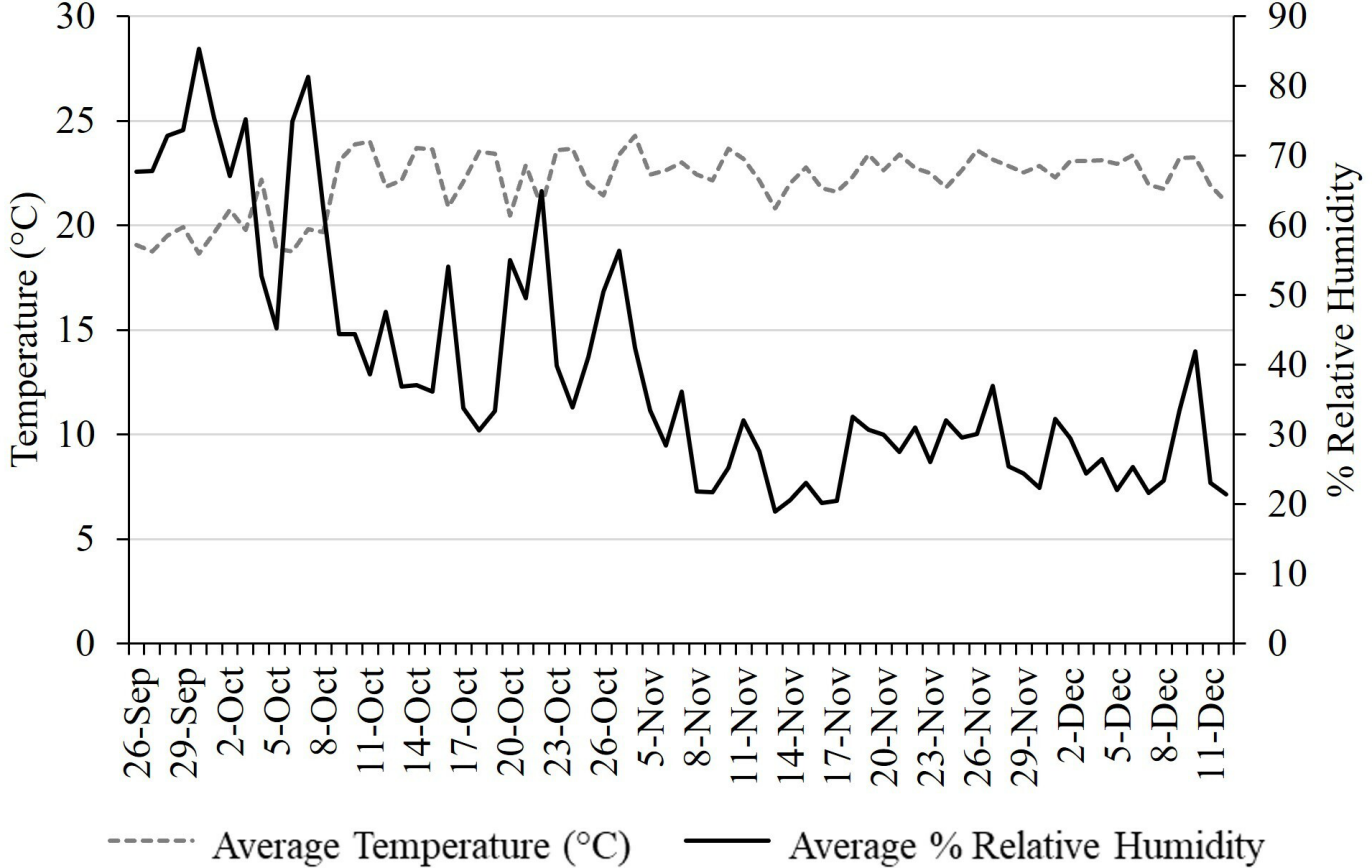
Daily temperature and humidity readings for L. delicatula oviposition study conducted 26 September – 11 December 2019.

In September 2019, assessment of abiotic conditions necessary to elicit successful mating and oviposition in the quarantine greenhouse and growth chambers was conducted. Sources of adults used in these trials included: 1) adults reared from field-collected eggs on *A. altissima* trees (1 cage, 17M: 18F), 2) adults reared from late instar (3^rd^ and 4^th^) nymphs collected from the field in Winchester, VA (1 cage, 19M: 20F), and 3) adults collected from the field in Winchester, VA (5 cages, 20-25 insects per cage, 1M: 1F). Cages containing adults from field-collected eggs and from late-instar nymphs were held in an environmental chamber at 12L:12D and 24°C:13°C to simulate natural conditions in late summer and fall. Cages containing field-collected adults were held within the greenhouse space underwent temperature and humidity as shown in **Figure 3** and natural light conditions, which ranged from 12L:12D in mid-September to 9.75L:13.25D at the end of November. Each cage (W47.5 x D47.5 x H93.0 cm, 680 µm aperture mesh, BugDorm-4S4590 Insect Rearing Cage, MegaView Science Co., Taiwan) was provided with the following substrates to promote egg laying: one potted *A. altissima* and one potted *V. riparia* (also serving as food sources), one *A. altissima* log (approx. 30 cm length, 8** **cm diameter), one red maple, *Acer rubrum* L. (Sapindaceae: Sapindales) log (approx. 30 cm length, 8** **cm diameter), and an open sided box constructed from two sheets of balsa wood (15 x 15 cm) separated by 2 cm spacers. Every 2-3 d, cages were inspected for fresh egg masses and dead adults were recorded and removed. Oviposition data were recorded from 26 September – 12 December 2019, concluding when the final female died. Data collected included oviposition date, substrate used, and number of egg masses per live female.

In 2020, adults produced from the two cohorts of nymphs were moved to cages containing their respective plant diets within an environmental growth chamber. This chamber was maintained at 65 ± 5% RH, 16:8 L:D, and 24 ± 3°C from July – September. From 1 October – November, the temperature was reduced to 18 ± 3°C but all other parameters remained the same. Each cage contained ~ 20 adults (~1M: 1F ratio) and was provided with one *A. altissima* log (approx. 30 cm length, 8** **cm diameter); the first cohort of nymphs resulted in one cage of adults from each diet combination and the second cohort of nymphs resulted in three cages of adults from each diet combination. Cages were inspected for new egg masses every 2 – 3 d and dead adults were recorded and removed.

### *Lycorma delicatula* survivorship and development from no-chill egg masses

A subset of the egg masses deposited on *A. altissima* logs in Fall 2019 were held in an environmental chamber at 12L:12D and 24°C:13°C with logs propped up diagonally within a cage (W32.5 x D32.5 x H77.0 cm, 680 µm aperture mesh, BugDorm) that contained a single potted *A. altissima* plant. After nymphs hatched on 2 January 2020, logs were removed, and chamber conditions were changed to 65% RH, 16:8 L:D, and 25°C, and a *V. riparia* plant was added. Survivorship and development of the hatched *L. delicatula* were monitored in this cage every 3 – 4 d until all insects died; plants were replaced when wilting occurred. As this was a preliminary trial, we did not record the total number of egg masses on logs or eggs per egg mass, therefore we provide descriptive results of documented egg hatch under no-chill conditions and nymphal development from hatched eggs.

## Results

### *Lycorma delicatula* development and survivorship on *Ailanthus altissima* diet preparations

**Figure 4:**
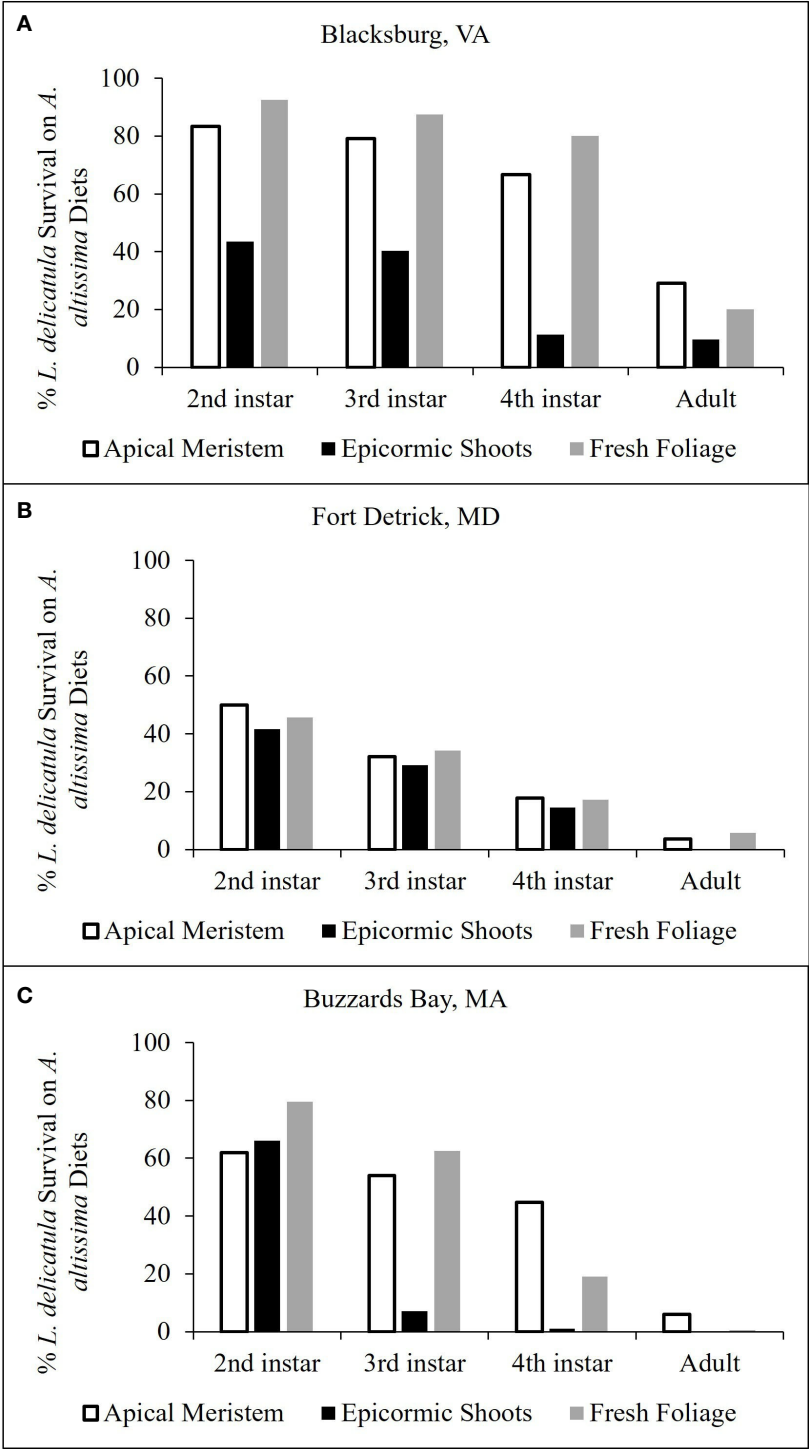
Percentage of surviving L. delicatula for each lifestage when 1st instars were reared on **(A)** altissima apical meristem, epicormic shoots, and fresh foliage. Results presented from experimental trials conducted in Blacksburg, VA **(A)**, Fort Detrick, MD **(B)**, and Buzzards Bay, MA **(C)**.

Across all cut *A. altissima* plant material diets, a higher percentage of *L. delicatula* survived and developed on apical meristem compared with epicormic shoots or freshly cut branches (**Figure 4**). For trials conducted in VA quarantine facilities, 20% developed to the adult stage on both apical meristem and freshly cut branches, with 9.5% reaching adulthood on epicormic shoots (**Figure 4A**), which resulted in a total of 21 *L. delicatula* adults from 126 hatched nymphs across all treatments. Trials conducted in MD and MA quarantine laboratories yielded no adults from the epicormic shoot diet, and those fed either apical meristem or freshly cut branches yielded fewer than 10% adults from the nymphal cohort (**Figures 4B, C**); these trials yielded 4 and 10 adults in the MD and MA facilities, respectively. In a pre-trial using potted *A. altissima* as a food source in MD quarantine facilities, 33 adults were produced.

### *Lycorma delicatula* development and survivorship on potted *A. altissima* and *V. riparia* plant diets

**Figure 5:**
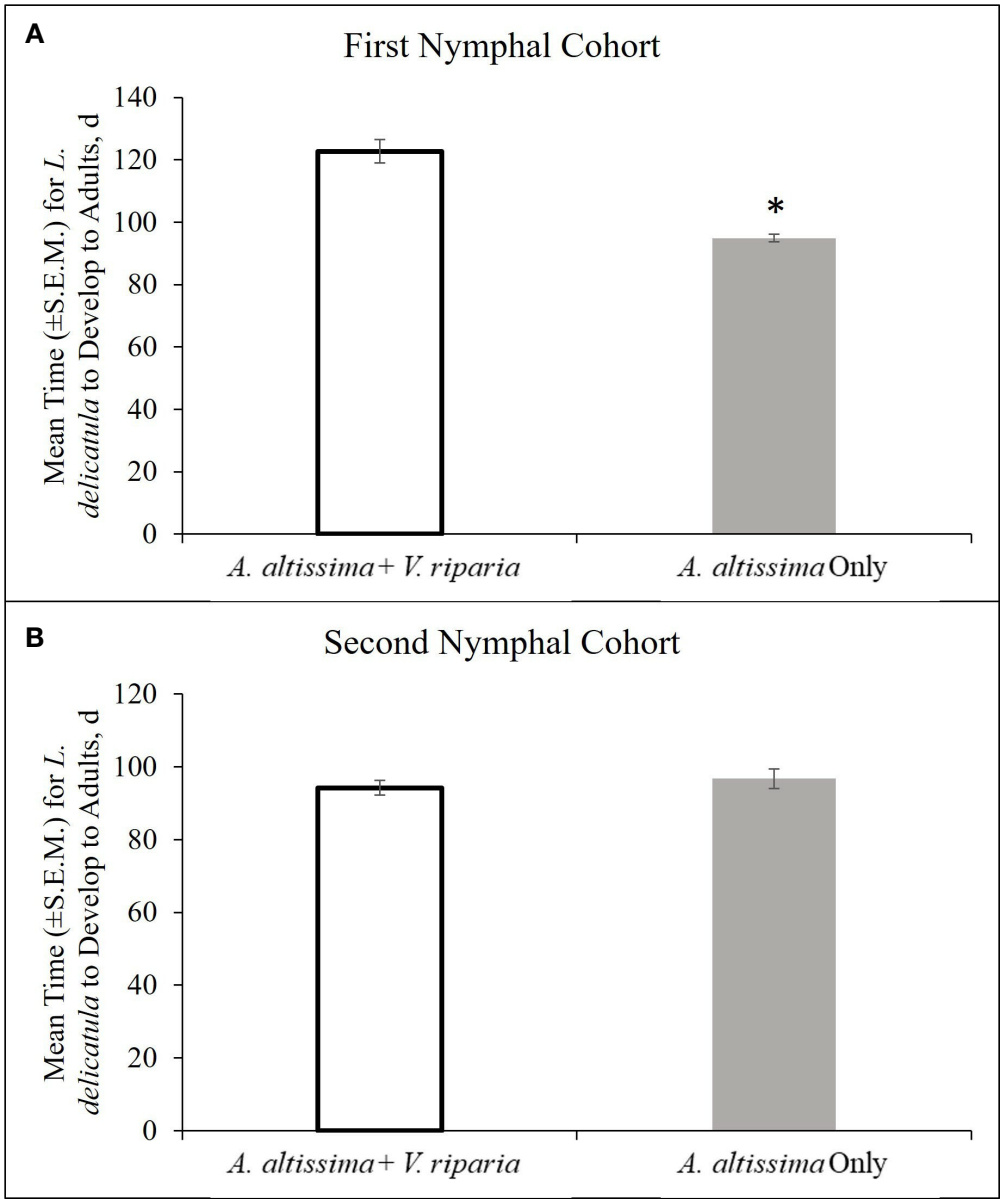
Developmental time (d ± S.E.M.) for L. delicatula from the first nymphal cohort (24 January – 8 May 2020) **(A)**, and second nymphal cohort (18 May – 10 September 2020) **(B)**.

The first nymphal cohort developed to the adult stage in a significantly shorter period of time on *A. altissima* plants alone compared with those reared on mixed diets of *A. altissima* and *V. riparia* plants (t = -6.96; df = 69.9; *P* < 0.001) (**Figure 5A**). The single host diet also had a significant effect on developmental time. Nymphs reared on a single host diet spent more days in the 2^nd^ (t = 7.78; df = 285.7; *P* < 0.001), and 3^rd^ (t = 2.15; df = 184.8; *P* = 0.017), instar lifestages and fewer days in the 4^th^ (t = -5.28; df = 83.3; *P* < 0.0001) instar lifetage compared to mixed diet reared nymphs (**Table 1**). The percentage of hatched nymphs that developed to the adult stage was similar for both diets with 26.6% on *A. altissima* with *V. riparia* (46 adults total) and 29.8% on *A. altissima* alone (50 adults total).

**Table 1:** Mean number d (+S.E.M.) spent by Lycorma delicatula in each nymphal lifestage when reared on A. altissima with Vitis riparia and A. altissima alone. * signifies significant difference in development time between diets.

Treatment	First Instar	Second Instar	Third Instar	Fourth Instar
	First Cohort			
Ailanthus altissima + Vitis riparia	20.4 ± 0.4	16.3 ± 0.3	20.7 ± 0.9	33.1 ± 1.9
Ailanthus altissima Alone	21.2 ± 0.4	19.7 ± 0.3*	22.8 ± 0.5*	22.1 ± 0.9*
	Second Cohort			
Ailanthus altissima + Vitis riparia	–	–	27.5 ± 1.1	38.1 ± 1.4
Ailanthus altissima Alone	–	–	22.2 ± 0.7*	45.8 ± 1.7*

For the second cohort, there was no significant difference in development time from hatch to adult between those reared on diets of *A. altissima* alone and those on *A. altissima* with *V. riparia* (t = 0.76; df = 107.6; *P* = 0.77) (**Figure 5B**). Diet had a significant effect on developmental time of nymphal instars. Nymphs spent fewer days in the 3^rd^ (t = 4.20; df = 149.7; *P* < 0.0001) instar lifestage and more time in the 4^th^ (t = 3.51; df = 111.7; *P* < 0.001) instar lifestage (**Table 1**). The percentage of hatched nymphs that developed to adults was comparable on both diets and greater than the first cohort: 59.8% on *A. altissima* with *V. riparia* (64 adults total) and 52.8% on *A. altissima* alone (57 adults total).

### Conditions necessary to elicit oviposition

**Figure 6:**
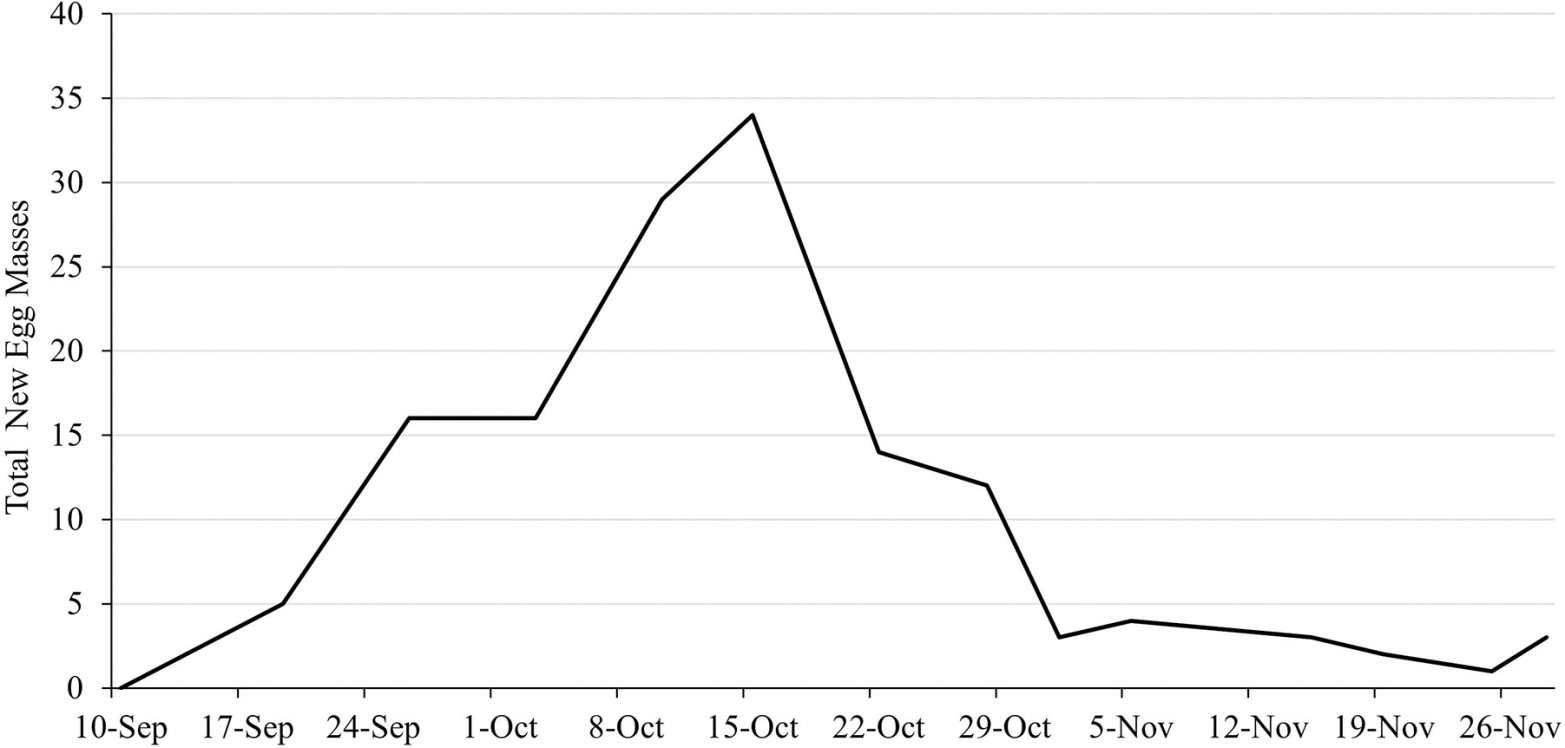
Non-cumulative phenology of egg mass deposition by female L. delicatula 10 September – 26 November 2019.

In 2019, a total of 133 egg masses were deposited by females, with each female depositing between 1-3 egg masses, for an average of 2.12 egg masses per female. Among substrates, 51% of all egg masses laid were deposited on *A. altissima* logs, 18% on *A. rubrum* logs, 19% on potted grape plants (principally on vines), and 12% on either the balsa wood structure or the cage structure itself. No egg masses were deposited on the live *A. altissima*. Oviposition began on 10 September and continued until 29 November with peak oviposition occurring in mid-October with 34 egg masses laid over a 4-day period (**Figure 6**).

In 2020, two egg masses were deposited in a cage containing four females with *A. altissima* and *V. riparia* (both on 19 October) and 10 egg masses were laid over two cages containing a total of 20 females with *A. altissima* alone from 4 November – 1 December, resulting in 0.5 egg masses per female for both diet treatments.

### *Lycorma delicatula* survivorship and development from no-chill egg masses

A total of 13 nymphs emerged from a single egg mass under no chill conditions. Of these, five *L. delicatula* developed to the adult stage: four males and one female. The first male emerged 84 d after hatch and the female 102 d after hatch. The female adult survived for 88 d and did not oviposit. The male adults survived 40 ± 14 d.

## Discussion

Here we demonstrated that *L. delicatula* can be reared from newly hatched first instar nymphs through to the adult stage under laboratory conditions using potted *A. altissima* trees while other *A. altissima* diet preparations did not result in reliable development to the adult stage. This approach is similar to rearing techniques for other insect species that require active vascular tissue for feeding, e.g., glassy-winged sharpshooter, *Homalodisca vitripennis* Germar (Hemiptera: Cicadellidae), with colonies provided host plants that generally need weekly replacement (18).

We also found that by reducing daylength from 16L:8D to 12L:12:D and providing *A. altissima* logs as a substrate, adult females would reliably oviposit under laboratory conditions. Indeed, females provided with these shorter daylength conditions to mimic those found in nature from mid-September onward deposited 4× as many eggs as those held at typical 16L:8D long-day conditions often used for standard insect colony maintenance. As *L. delicatula* is univoltine, and eggs are the overwintering lifestage, daylength appears to be an important cue for eliciting oviposition. Eggs are coated with a waxy material that provides an apparent barrier for protection (19), and females may be unwilling to oviposit too early to ensure eggs remain intact and well-protected. Moreover, as eggs are typically deposited on natural substrates, with *A. altissima* being the most common natural substrate (5), providing natural substrates in our rearing system appeared to also be critical to eliciting oviposition. Most eggs were deposited on *A. altissima* logs, though eggs were also deposited on *V. riparia* grapevines and *A. rubrum* logs.

Conversely, nymphal cohorts likely require longer daylengths to complete development. In the 2020 rearing experiments, the second cohort of nymphs had twice as many *L. delicatula* develop into adults than the first cohort; both cohorts were held at comparable temperatures, 20 – 25°C, but the first cohort was hatched and reared January – May (9.5L:14.5D – 14.5L:9.5D) and the second cohort during the time of year when wild *L. delicatula* in the region develop, May – September (14.5L:9.5D – 12L:12D). This would suggest that the natural daylengths of late spring and summer during this period is beneficial to nymphal development, particularly when compared with winter and early spring conditions. Degree day studies show that all mobile lifestages of *L. delicatula* survive and develop with temperatures between 15 and 30°C, and developmental rates increase with temperature within that range (20). Our rearing studies also show that longer daylengths are needed to support nymphal development, and variable humidity within that range did not seem to have a negative impact.

*Lycorma delicatula* egg masses undergo a prolonged period of chilling in nature throughout the winter months, although diapause requirements for *L. delicatula* are under-studied and likely include daylength as a vital cue. However, egg masses can be held at a constant 15°C with no chill period resulting in >50% hatch with comparable results to when egg masses were held at 10°C for an 84 d chill period and moved to 25°C for hatching (17). Thus, it does not appear that a chilling period (≤10°C) is required for *L. delicatula* embryo development. A study has shown that a 7 d chill period (5 or 10°C) is not sufficient for most *L. delicatula* eggs to fulfil diapause requirements, although a small number were able to develop (17). Recent unpublished data (MK) has shown that holding eggs at an alternating temperature regime mimicking a colder climate can delay hatch of fall collected eggs until June, resulting in ~70% egg hatch and more rapid nymphal development at lower temperatures (when compared to nymphs hatched at 15°C). Use of the alternating regime can prolong the period egg masses remain viable and provide hatch to work with later in the year. In our studies, egg masses held at 12L:12D and 24C:13°C yielded limited *L. delicatula* hatch. While this was relatively rare, it does raise the possibility that *L. delicatula* could establish in regions that were previously deemed unsuitable due to a lack of environmental chilling (21).

Although it is well established that *L. delicatula* exhibit a broad host range during earlier lifestages and a narrower range during late instar and adult stages (2, 22), *A. altissima* appears to be a preferred host throughout their development (8, 22). However, both greenhouse and field cage experiments have demonstrated that *L. delicatula* can develop on other hosts without the presence of *A. altissima*. In large field cages, *L. delicatula* have successfully developed to adulthood and reproduced when provided with weeping willow *Salix babylonica* L. (Malpighiales: Salicaceae), silver maple *Acer saccharinum* L. (Sapindales: Sapindaceae), and river birch *Betula nigra* L. (Fagales: Betulaceae) (7, 9). *Lycorma delicatula* have successfully completed development to the adult stage on single host diets of *J. nigra*, black walnut, and *Vitis vinifera* L. (Vitales: Vitacae) (Elsensohn et al. in prep., 11) in greenhouse trials. Interestingly, survivorship and development varies among *Vitis* spp. Here, we included *V. riparia* which had no real impact on survivorship and development of *L. delicatula* when combined with *A. altissima* compared with a diet of *A. altissima* alone. In other studies, *V. rotundifolia* could not support development or survivorship of *L. delicatula* as a single host diet, and its inclusion with *A. altissima* seemed to have no impact (11). However, when *V. vinifera* is used as a single host, development and survivorship to the adult stage occurred, and survivorship increased when combined with *A. altissima* (Elsensohn et al. in prep). Thus, host diet selection is critical to any rearing system for *L. delicatula*, and in this case, *V. vinifera* is the only *Vitis* species that supports strong development and survivorship.

## Summary

**Figure 7:**
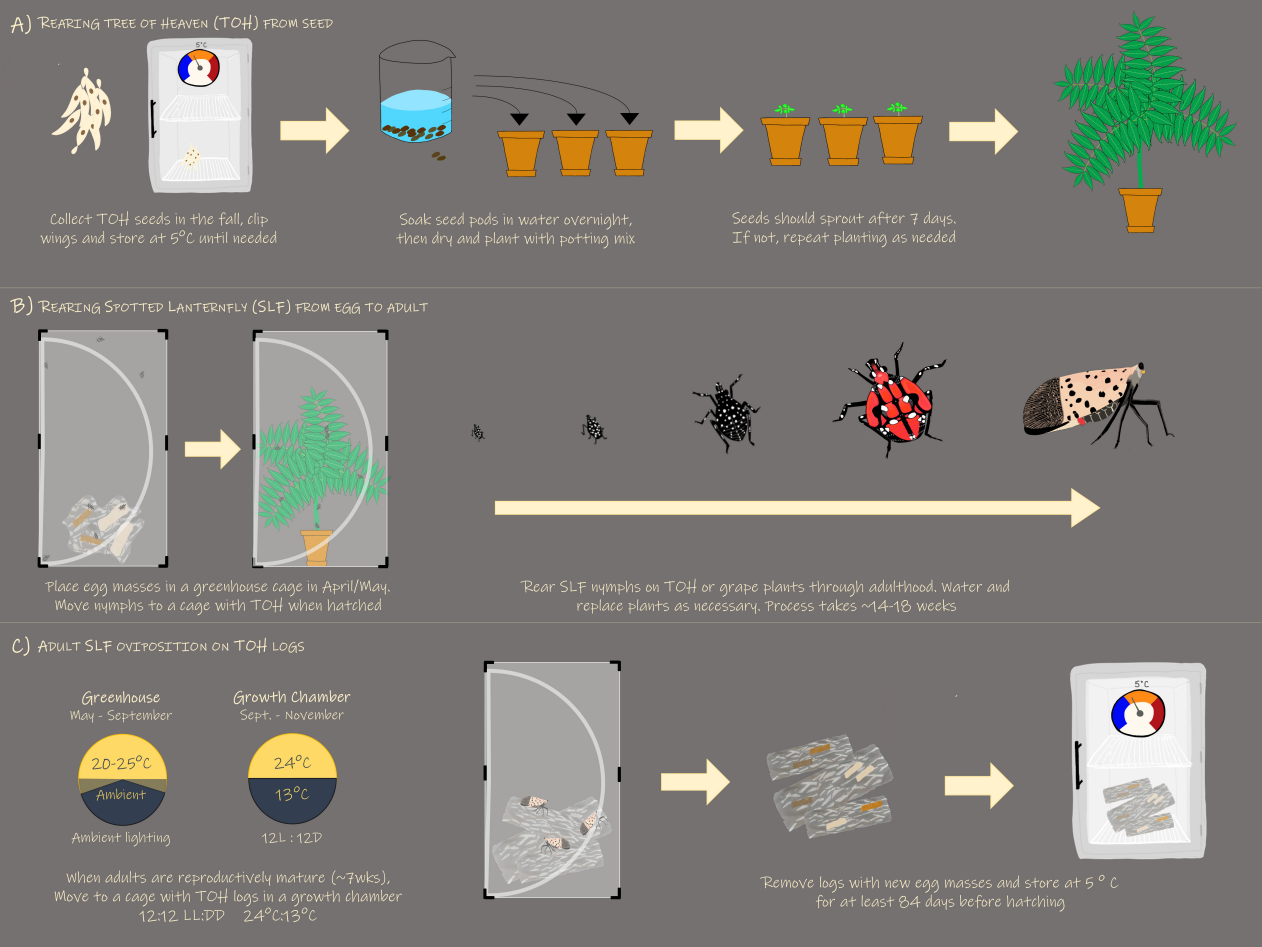
Graphic displaying the optimized method for rearing L. delicatula including **(A)** growing A altissima from seed, **(B)** rearing SLF from egg to adult, and **(C)** eliciting SLF oviposition. .

To successfully rear *L. delicatula* in the laboratory or greenhouse (**Figure 7**), eggs collected in the fall prior should be held at a constant 15°C for 90 – 100 d and eggs collected following significant chilling should be held at 10°C for 60 – 80 d. (17). To promote hatch, up to 15 egg masses should be placed in a typical insect rearing cage and provisioned with at least one 30 cm height potted *A. altissima* at 25°C and 16L:8D. Egg hatch should begin in ~7 – 14 d. *Ailanthus altissima* can be grown from stratified seeds germinating in ~4 weeks at 25°C, with subsequent potted plants reaching the appropriate 30 cm height in 8 – 10 weeks at ambient greenhouse conditions. Once hatch has occurred, 30 nymphs should be transferred to new cages containing a single *A. altissima*. Plants should be replaced at approximately 1–3-week intervals based on lifestage with 4^th^ instar nymphs and adults requiring every one to two weeks. Temperatures should be maintained at ~20–25°C at daylengths natural to late spring and summer months. To elicit mating and oviposition, adults should be maintained in cages with no more than 20 individuals and provisioned with an *A. altissima* plant as a food source and bolt as an oviposition substrate at 12L:12D and 24°C:13°C.

## Data availability statement

The datasets presented in this study can be found in online repositories. The names of the repository/repositories and accession number(s) can be found below: https://figshare.com/projects/Development_of_rearing_techniques_for_spotted_lanternfly/146295.

## Author contributions

All authors conceived, facilitated, and designed the research. SJ, AD, DL, MH, LS, and LN conducted the experiments. LN analyzed the data and conducted statistical analyses. LN and TL wrote the manuscript. TL, TK, JG, MK, and DP secured funding. All authors read and approved the manuscript.

## Funding

This work is supported by the National Institute of Food and Agriculture, U.S. Department of Agriculture, Specialty Crop Research Initiative under award number 2019-51181-30014. This work was funded in part by USDA APHIS PPQ S&T interagency agreement 10-8130-0840-IA (FS 19IA11242303103) with the Forest Service.

## Acknowledgments

Thank you to Morgan Douglas, Jessica Patterson, and Caitlin Barnes for excellent technical assistance. Mention of trade names or commercial products in this publication is solely for the purpose of providing scientific information and does not constitute recommendation or endorsement by the United States Department of Agriculture. USDA is an equal opportunity provider and employer.

## Conflict of interest

The authors declare that the research was conducted in the absence of any commercial or financial relationships that could be construed as a potential conflict of interest.

## Publisher’s note

All claims expressed in this article are solely those of the authors and do not necessarily represent those of their affiliated organizations, or those of the publisher, the editors and the reviewers. Any product that may be evaluated in this article, or claim that may be made by its manufacturer, is not guaranteed or endorsed by the publisher.
